# Determining the Lowest Optimally Effective Methotrexate Dose for Individual RA Patients Using Their Dose Response Relation in a Tight Control Treatment Approach

**DOI:** 10.1371/journal.pone.0148791

**Published:** 2016-03-17

**Authors:** Sandhya C. Nair, Johannes W. G. Jacobs, Marije F. Bakker, Z. Nazira Jahangier, Johannes W. J. Bijlsma, Jacobs M. van Laar, Floris P. J. G. Lafeber, Paco M. J. Welsing, Utrecht Arthritis Cohort Study Group

**Affiliations:** 1 Department of Rheumatology & Clinical Immunology, University Medical Center Utrecht, The Netherlands; 2 Department of Rheumatology, Tergooi hospital, Hilversum, The Netherlands; Nippon Medical School Graduate School of Medicine, JAPAN

## Abstract

**Objective:**

To determine the optimal methotrexate dose in individual patients and to explore whether this optimal dose and the level of disease activity at that dose could be predicted.

**Methods:**

Data from CAMERA II trial comparing MTX and MTX with 10 mg of prednisone both in a tight control treatment strategy in early RA was used. For each patient a curve for disease activity over time was fitted and the MTX dose after which further step-up did not result in relevant improvement in disease activity anymore was determined the 'lowest optimally effective MTX dose (LOED)'. The association of demographic and clinical characteristics at baseline with this LOED and with the level of disease activity reached at LOED was studied.

**Results:**

In 204 (100 MTX and 104 MTX with prednisone) out of 236 patients LOED could be defined. 10 mg/wk was the most prevalent LOED in patients treated with MTX and prednisone and 10 mg/wk, 20 mg/wk and 30 mg/wk in the MTX strategy. Although the specific LOED could not reliably be predicted, higher baseline disease activity, height and lower weight were associated with higher LOEDs (i.e at least 15 mg/wk). A score was presented to decide on a starting dose of 10 mg/wk or (at least) 15 mg/wk. The level of disease activity at LOED could not be reliably predicted.

**Conclusion:**

A starting dose of 10 mg/wk might be a good choice for most patients and is frequently already the optimal dose. However, a subgroup of patient can be determined who would require higher MTX doses.

## Introduction

In early rheumatoid arthritis (RA), intensive therapy immediately after disease onset nowadays is advocated to control disease activity as soon as possible. Preferably treatment should be started within the supposed ‘window of opportunity’, preventing early (progression of) radiographic joint damage, and reducing functional limitations.[1,2] Methotrexate (MTX) is the disease modifying anti-rheumatic drug (DMARD) of first choice in early RA. [1, 3–6] The drug is used in several step-up and step-down treatment strategies, often in combination with conventional and biological DMARDs and/or prednisone. [1, 7, 8] According to guidelines, in tight-control and treat-to-target strategies in early RA, the MTX-dose should be stepped-up rapidly. However, there is no generally accepted starting dose or dose steps defined.[3, 4] Tight control can be defined as a treatment strategy, aimed at the specific target of low disease activity or preferably remission (‘treat to target’) in an individual patient. This target is to be achieved within a reasonable period of time, preferably within the so-called ‘window of opportunity’. For this purpose disease activity is frequently monitored and treatment is adjusted accordingly. [9–13]

Tight control strategies with predefined dose and strategy steps and protocolized treatment adjustments have on group level been found to be more effective than conventional treatment strategies.[13] In clinical practice, maintenance doses of MTX range from 7.5 to 30 mg/wk, suggesting that different patients might benefit from different dosing for optimal disease control. Prediction of response to MTX has not yet resulted in guidance for the initiation of MTX or the optimal starting dose, or optimal maintenance dose of MTX for individual patients. [14–17] Therefore and for dose-dependent safety issues, in clinical practice, the MTX dose is usually stepwise increased starting at a low (often ineffective) dose, and increasing the dose until the treatment target is reached or toxicity occurs: a step-up strategy. This means in general an (temporary) overdose, as increments with the next dose step might not result in clinically relevant improvement anymore. In case the treatment target is not reached, this would mean a delay in taking further, more effective strategy steps (i.e. under treatment), such as adding another DMARD. Similarly, a protocolized low starting dose of MTX could be too low for certain patients, also leading to under treatment because of unnecessary delay to reach optimal disease control.

Another strategy might be to start with a high dose and to decrease the dose stepwise when the disease is sufficiently controlled: a step-down strategy. Also for this strategy, it would be of value to know the lowest dose of MTX which is optimally effective in controlling disease activity in individuals (i.e. the dose at which a further dose decrease will lead to a clinically relevant increase in disease activity). The ‘lowest optimally effective dose’ (LOED) could serve as a starting dose or a dose to reach as soon as possible and/or as a dose after which other treatment strategy steps (i.e. combining or switching therapy) should be considered if disease activity still is too high. It would also be valuable to know the level of disease activity that can be reached at this LOED. For instance, if sufficient disease control in an individual is not to be expected with MTX monotherapy at LOED, right from the start of treatment an MTX-based combination (DMARD) strategy might be considered. Prediction of the LOED for individual patients may add to personalized medicine, and optimal use of the window of opportunity in treatment.

The aim of the current study was to determine (variation in) the LOED, to predict disease activity at the LOED, and LOED itself.

## Patients and Method

The systematic increase of the dose of MTX monotherapy from 10 mg/wk to 30 mg/wk in the Computer Assisted Management in Early Rheumatoid Arthritis (CAMERA) II trial comprised a good opportunity to study the dose response relation of MTX and thus to determine LOED in individual patients. [5]

Within the CAMERA II trial, the effects of an MTX-based, tight control strategy starting MTX with 10 mg of prednisone (MTX+ pred) and MTX with placebo (MTX) were compared. In both strategies oral MTX was up-titrated from a dosage of 10 mg/wk to 30 mg/wk (or the maximum tolerable dose). If 30 mg/wk of MTX (or the maximum tolerable dose) was reached without reaching the treatment target, the next step in the treatment strategy was taken. It was found that the treatment strategy using MTX along with prednisone was more effective in reducing disease activity over the 2 years of the trial and more patients reached a state of remission (72% in MTX + pred and 61% in MTX strategy, respectively). [5]

For the current analysis, the data of all patients from the CAMERA II trial (n = 236) was used for the time period they were on oral MTX monotherapy with or without prednisone, i.e. the time from start of treatment until they started the next strategy step, or dropped out of the study.[5] The disease activity score based on 28 joints (DAS28) was used as measure of disease activity. [18] Further, data on age, gender, rheumatoid factor (RF) status, body weight, height, serum creatinine, Alanine Transaminase (ALT) and Aspartate Transaminase (AST), and functional disability (Health Assessment Questionnaire, HAQ) was available and used.

### Determination of Lowest Optimally effective MTX Dose (LOED)

To estimate the course of the disease activity over time within each patient, DAS28 measurements over time were plotted. As disease activity can fluctuate much over time, representing actual fluctuation and measurement error, changes based on actual disease activity measurements (to determine LOED) would probably misclassify the actual course of disease activity over the time period quite often. To control for this (physiological and random) variation in DAS28 a ‘power curve’ (a non-linear model) was fitted through the actual DAS28 measurements over time to attain a smooth curve of disease activity over time for each individual patient. To fit this curve the procedure NLIN in the statistical software package SAS (version 9.1) was used to fit a curve of the form p_1_*p_2_^time^ + p_3_ in which p_1_ can be regarded as the level of disease activity at baseline (i.e. at baseline p_2_^time^ becomes 1), p_2_^time^ represents the exponential decrease (where p<1) in disease activity over time, and p_3_ can be regarded as the level of disease activity reached (i.e. with high values of time, p_1_*p_2_^time^ approaches 0).

**Fig 1:**
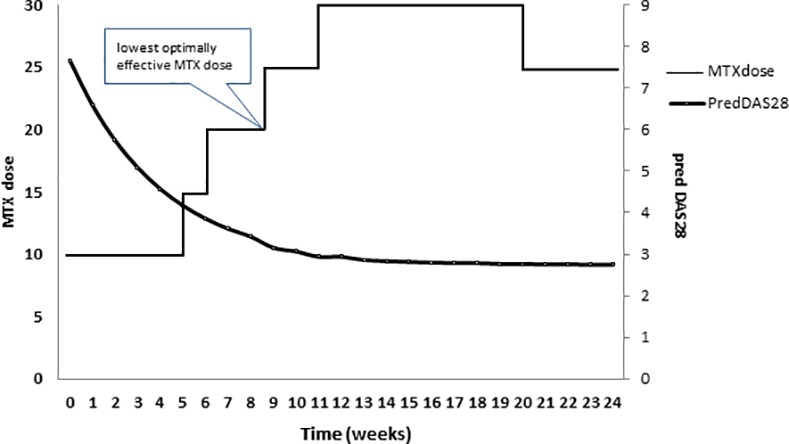
Example of one patient’s determination of LOED based on MTX doses over time along with estimated DAS28.

For every visit the DAS28 and improvement in the DAS28 according to this curve were determined for every patient. The highest MTX dose which still resulted in a clinically relevant improvement in predicted DAS28 was defined as the LOED. The threshold for clinical relevant improvement was defined as an improvement in DAS28 of 0.15 as compared to the previous visit [19]. This threshold, significantly smaller than the proposed minimal important difference in DAS28, was chosen not to miss small important improvements. If a patient never improved clinically relevantly or if a patient was still relevantly improving on the last MTX dose step, LOED could not be determined. **Fig 1** shows an example where we determined LOED (20 mg) for a patient based on the course of smoothed DAS28 and MTX dose over time.

The fit of the individual curves of predicted DAS28 over time to the observed DAS28 values was investigated graphically, by comparing predicted and observed DAS28 values and checking for systematic differences between observed and predicted values.

LOEDs were described and compared to the highest actually given MTX dose, to detect overtreatment. To detect possible under treatment, the time period until LOED was reached (i.e. time on suboptimal MTX dose) and the disease activity score reached were calculated.

### Prediction of disease activity at the LOED

Using multivariate linear regression, the effects of baseline clinical and demographic characteristics (including treatment strategy) and LOED were investigated at the level of disease activity reached at LOED.

### Prediction of LOED

Multinomial logistic regression analysis was used to study associations of demographic and clinical characteristics at baseline (including treatment strategy, i.e. addition of prednisone) with LOED (as nominal dependent variable). Based on the final model, for each patient the probability of having a certain LOED for all possible MTX doses (i.e. the probability that LOED is 15mg, 20 mg and so on for each patient) was calculated. The predictive properties of the model were investigated by comparing the predicted LOEDs (i.e. LOED with the highest predicted probability) of patients with the observed LOED.

Since this was a first attempt to look at a set of possible predictive factors for disease activity at LOED as well as LOED itself, univariate and multivariate regression analyses were performed using a manual backward selection strategy based on a p-value smaller than 0.2 and based on the effect on explained variance (for the linear regression analysis). A possible modifying effect of treatment strategy on the effect of other predictors was also investigated in both regression analyses.

## Results

**Fig 2:**
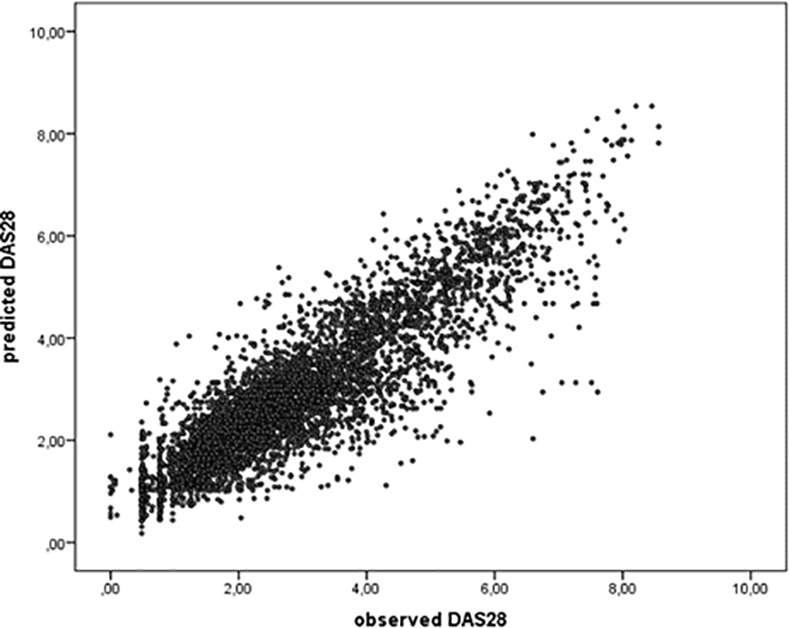
Fit of the predicted DAS28 to the observed DAS28.

Data of all 236 patients in the CAMERA II trial were used to fit a curve over time and calculate predicted DAS28 scores. On inspection of the dot-plots, the predicted and observed DAS28 values in general showed a good fit, indicating that the model adjusted adequately for the variability in disease activity (**Fig 2**).

Twenty two patients never had an improvement more than 0.15 in DAS28 (i.e. no clinically relevant improvement) and 10 patients were still improving clinically relevantly at the end of their follow-up; hence LOED was undefined in these 32 patients. Therefore for 204 (100 MTX and 104 MTX with prednisone) patients LOED could be defined.

**Table 1** shows the demographic and clinical characteristics of the patients for whom LOED could be calculated, overall and by treatment arm. Few differences in clinical and demographic characteristics between the strategy groups were observed. As expected due to the combination MTX with pred, the average highest MTX dose used was lower in the MTX + pred strategy and the EULAR response was higher. Overall, the baseline clinical and demographic characteristics of the current study are similar to the total CAMERA II population.

**Table 1:** Demographic and clinical characteristics including early response of patients for which LOED was determined per strategy arm.

Characteristics	Total	MTX	MTX+ pred	P-Value
	N = 204	N = 100	N = 104	
Age in years, mean (sd)	54 (13.6)	53 (12.9)	55 (14.3)	0.16
Female gender, n (%)	118 (57.8)	60 (60)	58 (55.8)	0.54
Rheumatoid factor positive, n (%)	163 (84.9)	82 (84.5)	81 (85.3)	0.89
DAS28 baseline, mean (sd)	5.7 (1.3)	5.6 (1.2)	5.7 (1.3)	0.30
HAQ baseline, median (Q1,Q3)	1.06 (0.53, 1.44)	1.11 (0.75, 1.56)	0.78 (0.33, 1.33)	0.002
Highest MTX dose (mg/wk), mean (sd)	23.2 (7.1)	25.6 (5.6)	20.8 (7.6)	< .0001
Highest MTX dose,10mg/wk n (%)	22 (10.8)	1 (1)	21 (20.2)	
Highest MTX dose,15mg/wk n (%)	33 (16.2)	10 (10)	23 (22.1)	
Highest MTX dose,20mg/wk n (%)	33 (16.2)	19 (19)	14 (13.5)	
Highest MTX dose,25mg/wk n (%)	23 (11.3)	13 (13)	10 (9.6)	
Highest MTX dose,30mg/wk n (%)	93 (45.6)	57 (57)	36 (34.6)	
Maximum follow-up, mean (sd)	24 (5.9)	24.3 (5.7)	23.5 (6.1)	0.34
EULAR response at 3 months				< .0001
Good, n (%)	80 (44)	24 (26.1)	56 (62.2)	
Moderate, n (%)	64 (35.1)	38 (41.3)	26 (28.9)	
None, n (%)	38 (20.9)	30 (32.6)	8 (8.9)	
Height in cms, mean (sd)	173 (9.7)	172.4 (9.7)	172.8 (9.6)	0.78
Weight (kg), mean (sd)	76.6 (13.7)	76.4 (13.7)	76.9 (13.9)	0.79
BMI, mean (sd)	25.7 (4.0)	25.9 (3.7)	25.6 (4.2)	0.66
Serum creatinine in μmol/l, mean (sd)	73.8 (14.4)	72.4 (13.1)	75.3 (15.5)	0.15
Creatinine clearance in ml/min),mean (sd)	90.9 (26.2)	94.1 (26)	87.5 (26.2)	0.10
AST in U/L, mean (sd)	22.8 (10.4)	22.8 (8.8)	22.7 (11.8)	0.98
ALT in U/L, mean (sd)	28.0 (21.8)	29.8 (22.1)	26.2 (21.3)	0.25

DAS28: 28 joint count disease activity score; HAQ: Health assessment questionnaire; BMI: body mass index; MTX: methotrexate; MTX+pred: MTX based strategy with prednisone; AST: aspartate aminotransferase; ALT: alanine aminotransferase, Q1, Q3: 25^th^ and 75^th^ percentile (interquartile range)

Overall, 10 mg/wk was the most frequently (40%) observed LOED (Table 2). In the MTX + pred group LOEDs were generally lower; 10 mg/wk was the most prevalent LOED in the MTX + pred strategy and 10 mg/wk, 20 mg/wk and 30 mg/wk were the most prevalent LOEDs in the MTX strategy with comparable frequencies. On average (SD), the optimally effective dose was 17.4 (7.6) mg/wk, this was 20.3 (7.5) mg/wk in MTX and 14.6 (6.7) in MTX + pred strategy, respectively. **(Table 2).**

**Table 2:** Lowest optimally effective MTX dose (LOED) reached per strategy arm.

Characteristics	Total	MTX	MTX+ pred	P-Value
	N = 204	N = 100	N = 104	
Number of patients at different LOEDs - 10 mg/wk, n(%)	82 (40.2)	21 (21)	61 (58.7)	< .0001
- 15 mg/wk, n(%)	35 (17.2)	18 (18)	17 (16.4)	0.866
- 20 mg/wk, n(%)	31 (15.2)	20 (20)	11 (10.6)	0.106
- 25 mg/wk, n(%)	19 (9.3)	15 (15)	4 (3.9)	0.012
- 30 mg/wk, n (%)	37 (18.1)	26 (26)	11 (10.6)	0.014
LOED, mean (sd),	17.4 (7.6)	20.3 (7.5)	14.6 (6.7)	< .0001
Time at LOED (months), mean (sd)	3.2 (2.4)	3.9 (2.3)	2.6 (2.3)	< .0001
Reached DAS28 at LOED, mean (sd)	3.1 (1.4)	3.2 (1.4)	3.0 (1.5)	0.27

The average (SD) DAS28 that was reached with the optimal MTX dose was 3.2 (1.4) in the MTX strategy and 3.0 (1.5) in MTX + pred strategy. Sixty two percent (62%) and fifty seven percent (57%) of patients in MTX with prednisone and MTX strategy respectively had been treated with a higher MTX dose than the calculated LOED, suggesting overtreatment. LOED was reached within about 3 months and about 4 months on average in the MTX with prednisone and MTX strategy group, respectively, however, with substantial variation between individual patients.

### Prediction of DAS28 reached at LOED

The results of the linear regression analysis with DAS28 reached at LOED as outcome showed a very low explained variance (R^2^ = 0.011) of the final model and therefore did not provide clinical relevant prediction (results not shown).

### Prediction of LOED

Using the multinomial regression analysis, no strong predictors could be established and predicting the specific optimal MTX dose for a patient for clinical practice was not possible (results not shown). We therefore performed an analysis to see whether it could be predicted if LOED was 10 mg/wk (most frequently occurring LOED) or a higher LOED would be indicated using binary logistic regression. In that last case a starting dose of 15 mg could be used (since LOED then would be at least 15 mg). Based on this simpler approach a model with reasonable discriminative ability could be derived (AUC 0.72, 95% CI 0.64 to 0.80). No modification of the predictive effect of variables by treatment strategy was identified. After internal validation of the regression model (using bootstrapping) and rounding of the regression coefficients, the following prediction score could be derived from this analysis: - 86–0.2 * (weight) + 0.3 *(height) - 17 * (MTX+pred treatment strategy) + 2 * (DAS28 baseline. When treatment strategy is MTX+pred the variable has the value 1 and if only MTX treatment is used it is 0. For instance for a patients with a weight of 61 kg and a height of 168 cm, a DAS28 of 4.6 on MTX treatment has a score of (-38.6) rounded to 39. Patients with a higher score than 95 based on this function were found to have a probability of 82% to require an MTX dose of at least 15 mg/wk for optimal effectiveness. Forty-seven percent (47%) of the study population was classified as requiring at least 15 mg/wk of MTX in this way.

## Discussion

The lowest optimal MTX dose (LOED), i.e. the MTX dose after which a further increase in dose does not further improve disease activity to a clinically relevant extent anymore in the individual patient, differs among individual early RA patients. However, 10 mg/week, often used as the dose to start, is already the optimally effective dose in many cases, especially when combination therapy (with prednisone) is used. The disease activity reached at LOED or the specific LOED itself could not be well predicted. However, a subgroup of patients needing higher MTX doses might be delineated according to our prediction score based on a patient’s disease activity, height, weight, and whether the start of mono-or combination treatment is planned. In these patients therefore 15 mg/wk MTX or more might be used as a starting dose to speed up the process of reaching optimal control of disease activity within the window of opportunity.

The current treatment approach for RA is to achieve remission or at least low disease activity as soon as possible. Based on our results, it is recommended to either start with 10mg/wk (as is generally done) or start with 15 mg/wk in a subgroup based on the presented score. In case of insufficient disease control, the MTX dose should be increased until no further clinically relevant improvement in disease activity is expected based on a careful evaluation of changes in disease activity over time or the maximum (tolerable) MTX dose is reached. When it is expected that disease activity will not improve sufficiently on MTX as single DMARD, starting with combination therapy, including e.g. glucocorticoids should be considered. [5] This approach should be recommended, rather than using a standard scheme of dose increments for all patients. This is also in line with and might extent recent guidelines for MTX treatment. [3, 4, 7, 8, 20]

It has been suggested that MTX and prednisone are both ideal anchor drugs for the treatment of RA which maximizes the benefit for patients to reduce long term risks.[21] Our analysis also confirms the findings by Bakker et al. [5] that addition of prednisone to MTX treatment in the early phase of the disease results in lower dose of MTX needed to control disease activity. In additional analyses, it was found that the addition of 10 mg prednisone daily to the methotrexate-based tight control strategy during the 2-year study had not led to bone loss. In both strategy groups which received also a bisphosphonate, calcium and vitamin D even a small increase in lumbar BMD during the first year of treatment was found. [22] With a treatment approach like tight control, carefully monitoring the changes in disease activity over time with increase in MTX dose for an individual patient might partly prevent increasing the MTX dose unnecessary before taking other treatment steps. The prediction score distinguishes a subgroup of patients who would benefit from 15 or more mg/wk of MTX from the start of treatment. It has also been suggested in a systematic review [4] that the starting dose for MTX should be 10 to 15 mg/wk with a rapid dose escalation to control the disease. However, identifying the patients who would require either 10 mg/wk or higher doses before the start of MTX therapy may (further) reduce over as well as under treatment.

A limitation of our study was not accounting the use of subcutaneous MTX. In the CAMERA II study, 86 patients (60 in MTX group, 26 in MTX+ pred group) started subcutaneous MTX. However, according to protocol this was only started after patients did not reach enough decrease in disease activity with an oral dose of 30mg/wk of MTX (or the highest dose tolerated). [5] Patients started subcutaneous MTX at the same dose as the oral MTX on average after 7 months and 78% (67) needed to proceed to the next treatment step (i.e. start adalimumab) within the timeframe of the study. Since subcutaneous MTX was only given after up-titration of oral MTX and was itself not systematically up- or down-titrated optimal dosing of subcutaneous MTX could not be determined.

Further in CAMERA II MTX dose was increased every month when predefined criteria for response were not met. The full effect of (a certain dose of) MTX might be not be fully apparent until 3 months. Although this might lead to an underestimation of LOED, we think in general an important part of the effect might already be present and the small threshold for a clinical important improvement might make this a limited problem. Further the specific criteria for increasing the MTX dose as used in CAMERA II (i.e. not meeting the response criteria: improvement >20% in swollen joint count and at least in 2 of the following: tender joint count, erythrocyte sedimentation rate, and visual analogue scale for general well-being, compared with the previous visit) might influence the observed DAS28 curve over time. However, we don’t think that this influenced our findings importantly since we used the fitted curve through the observed DAS28 values per patient and used a small threshold for clinical important improvement. Increasing or decreasing the threshold did not importantly influence our results.

Another limitation to the study was that LOED could not be established for some patients. These were patients who never had any clinically relevant change or who were still improving. These patients can therefore only be identified by carefully monitoring disease activity in response to MTX dose changes in these patients. Also the effectiveness of MTX might relate to race. In our study group the large majority of patients were Caucasian, making subgroup analyses not feasible; the race with the greatest difference in MTX-tolerability is Japanese; there were no participating Japanese. Finally our prediction score for prescribing either 10 mg/wk of MTX or higher dosages (i.e. at least 15 mg/wk) as a starting dose has not been validated in another population. This is still needed before widespread use of such a score.

In conclusion, a starting dose of MTX of at least 10 mg/wk seems a good general choice based on our results, especially in combination treatment (with prednisone). The MTX dose should be increased in a tight-control strategy until no further improvement in disease activity is expected based on the course of disease activity over time, or until the treatment target or maximum (tolerable) dose is reached. It might also be possible to distinguish a subpopulation that would benefit from starting with a higher MTX dose of at least 15 mg/wk. Using this higher starting dose, patients can reach optimal control of disease activity earlier within the window of opportunity.

## Key Messages

- Standard MTX dosing strategies might result in over treatment or under treatment in several patients.
- The lowest optimally effective MTX dose varies between individual patients, but was found to be at least 10 mg.
- However, for a subgroup of patient it could be determined that 15mg/wk was most beneficial and therefore a starting dose of 15 mg/wk might be a good choice for these patients.
- Systematically monitoring changes in disease activity after treatment steps over time in a tight control treatment strategy allows determining when to stop increasing the dose to optimize treatment for individual RA patients.
